# Supplementary material for: Associations of biological ageing and genetic risk with incident abdominal aortic aneurysm
Source: Commun Med (Lond). 2026 Jan 9;6:107. doi: 10.1038/s43856-025-01373-w (PMC12894926; doi:10.1038/s43856-025-01373-w)
Supplement: Supplementary file 2 — Supplementary Information [file 43856_2025_1373_MOESM2_ESM.pdf]

Associations of biological ageing and genetic risk with incident abdominal aortic aneurysm

Chen Yao, PhD #, Guochang You, MD #, Runnan Shen, MD #, Kangjie Wang, PhD , Yunhao Sun, MD , Xiong Chen, MD , Kai Huang, PhD *

Table of Contents

Figure S1. Histograms of KDMAge and PhenoAge acceleration in the study.........................2
Figure S2. Distribution of biological age acceleration stratified by abdominal aortic aneurysm status. 3
Figure S3. Cumulative risk of incident abdominal aortic aneurysm events stratified by biological ageing status. 4
Figure S4. Association of polygenic risk score and incident abdominal aortic aneurysm. 6
Table S1. Diagnosis codes used by the UK Biobank for determining the outcome and related medical history. 8
Table S2. Definition of a healthy diet score used in this study in the UK Biobank. 9
Table S3. Data field ID of variables used in the study in the UK Biobank. 10
Table S4. Range of biological age accelerations. 11
Table S5. Stratified analyses of the associations between biological ageing and the risk of incident abdominal aortic aneurysm. 12
Table S6. Association between PRS category and incident abdominal aortic aneurysm risk. 14
Table S7. Association between pack-years of smoking and incident abdominal aortic aneurysm risk. 15
Table S8. Association between pack-years of smoking and biological age acceleration. 16
Table S9. Mediation effect of biological age acceleration on the association between pack-years of smoking and abdominal aortic aneurysm. 17
Table S10. Association between biological age acceleration and incident abdominal aortic aneurysm risk after excluding participants diagnosed with AAA during the first two years of follow-up. 18
Table S11. Association between biological age acceleration and incident abdominal aortic aneurysm risk in participants without missing covariates. 19
Table S12. Association between biological age acceleration and incident abdominal aortic aneurysm risk among participants without self-reported poor health at baseline. 20
Table S13. Association between biological age acceleration and incident abdominal aortic aneurysm risk in the Fine and Gray competing risk model. 21

Supplemental Figures

Figure S1. Histograms of KDMAge and PhenoAge acceleration in the study.

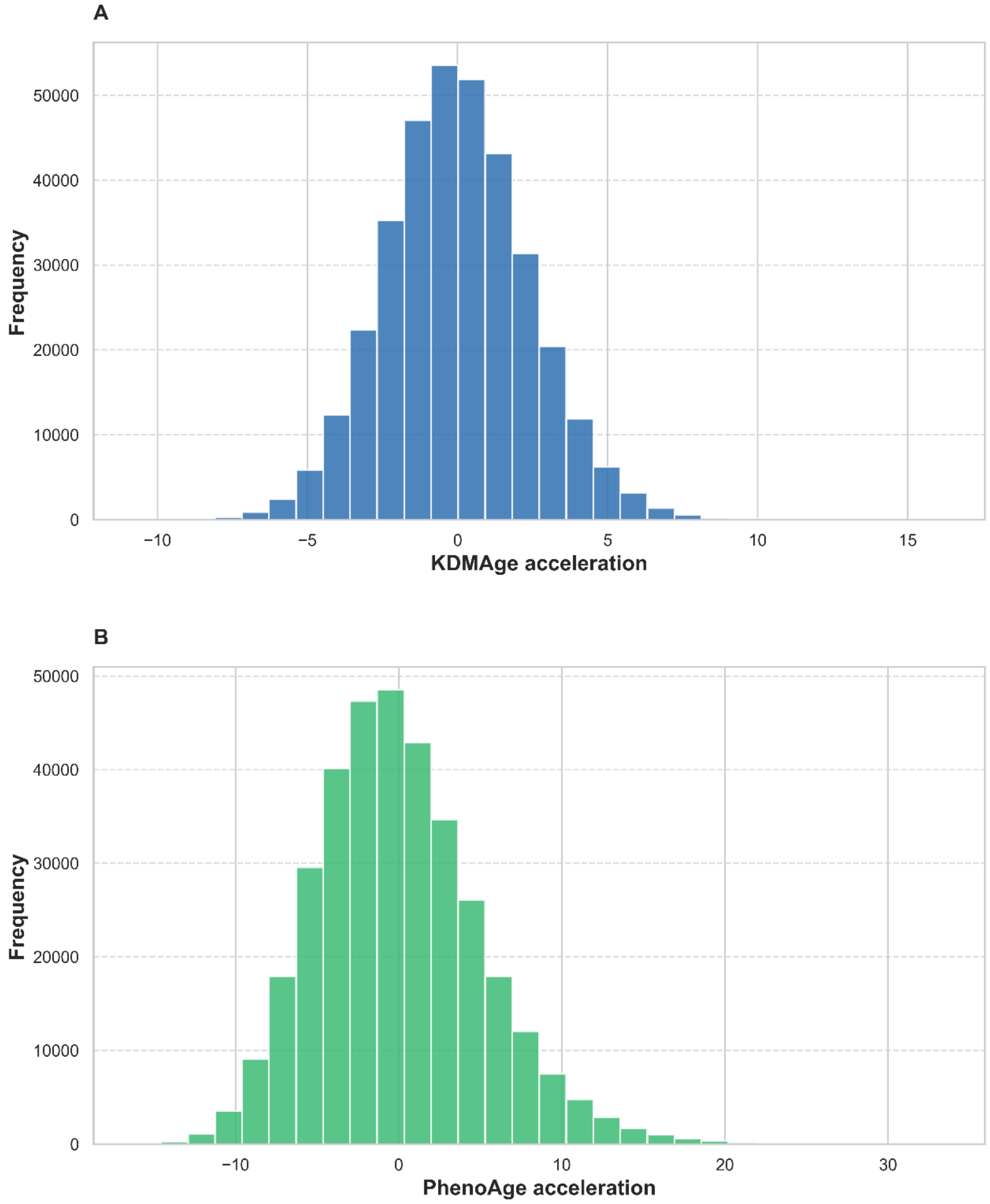

Histograms of (**A**) KDMAge acceleration and (**B**) PhenoAge acceleration in the study (n = 350,483). KDMAge, biological age calculated by the Klemera-Doubal method; PhenoAge, phenotypic age.

Figure S2. Distribution of biological age acceleration stratified by abdominal aortic aneurysm status.

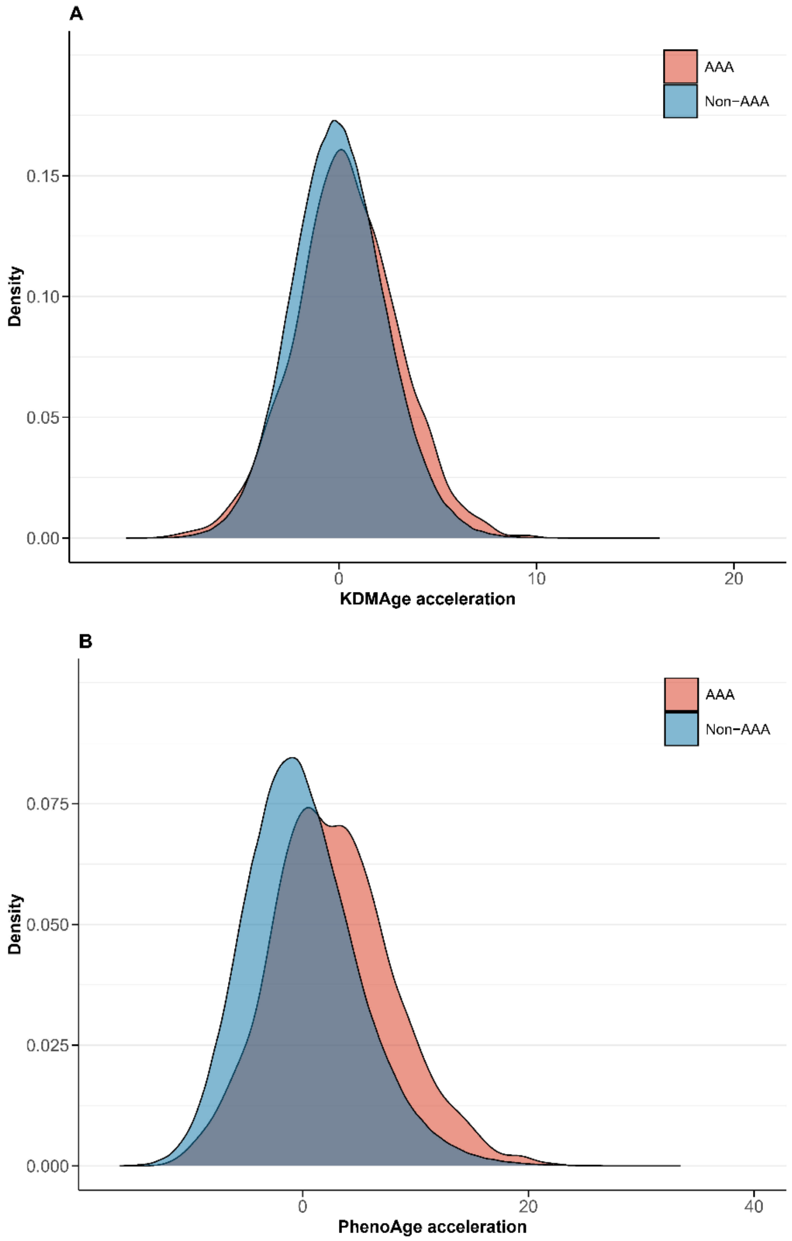

Distribution of (**A**) KDMAge acceleration and (**B**) PhenoAge acceleration by incident AAA status. AAA indicates abdominal aortic aneurysm; KDMAge, biological age calculated by the Klemera-Doubal method; PhenoAge, phenotypic age.

Figure S3. Cumulative risk of incident abdominal aortic aneurysm events stratified by biological ageing status.

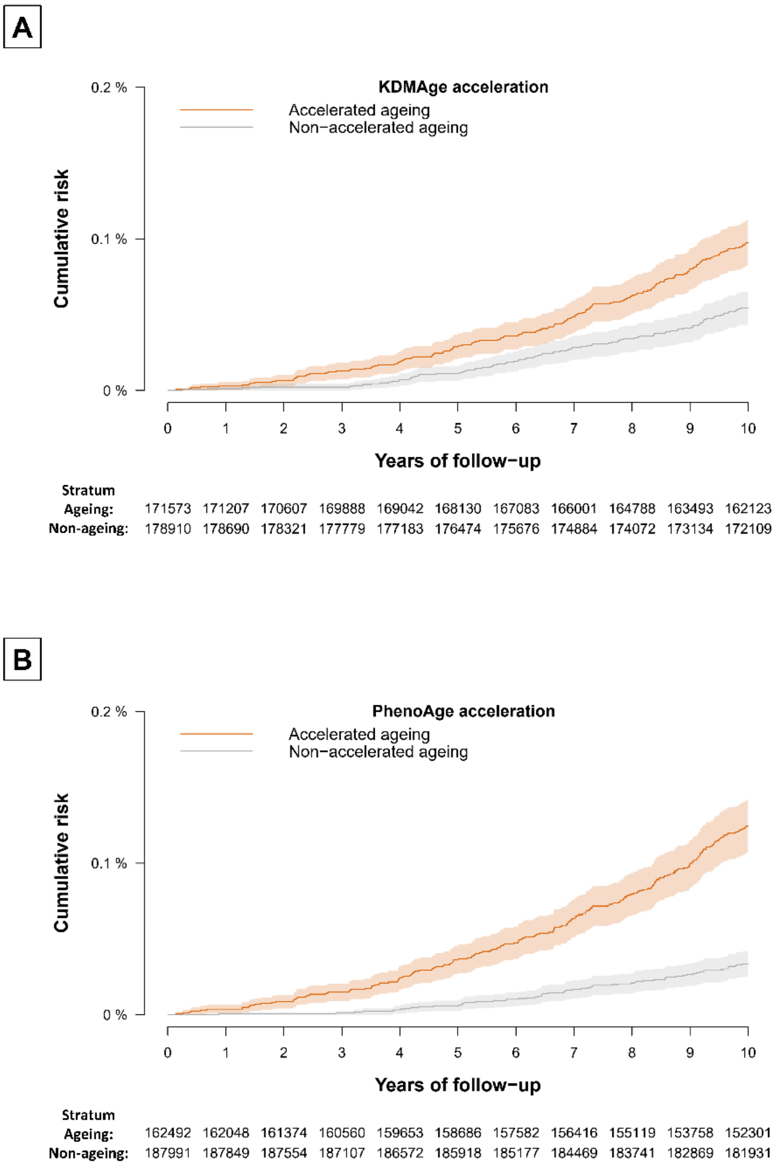

Plots depicting the crude cumulative risk of incident abdominal aortic aneurysm for (**A**) KDMAge and (**B**) PhenoAge acceleration, stratified by biological ageing status. Accelerated ageing indicates that the residual of the regression of KDMAge or PhenoAge based on chronological age is >0. Non-accelerated ageing indicates that the residual of the regression of KDMAge or PhenoAge based on chronological age is ≤0. The number at-risk over time is depicted below each plot. Shaded regions depict 95%

confidence intervals. KDMAge indicates biological age calculated by the Klemera-Doubal method; PhenoAge, phenotypic age.

Figure S4. Association of polygenic risk score and incident abdominal aortic aneurysm.

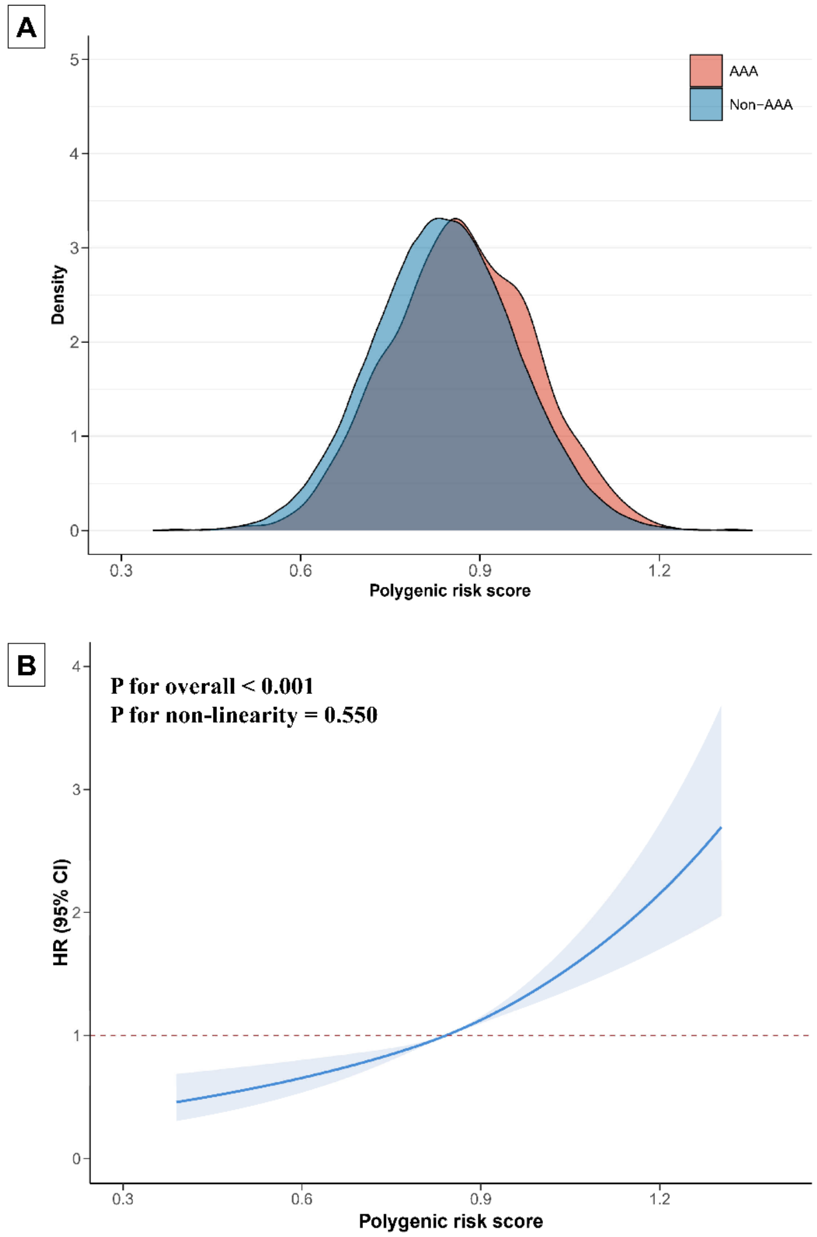

Plots depicting the (**A**) distribution of polygenic risk score (PRS) by incident abdominal aortic aneurysm (AAA) status, and (**B**) the dose-response association of AAA with the risk of incident AAA. Hazard ratios (solid lines) and 95% confidence intervals (shaded areas) were adjusted for age, sex, body mass index, education, employment, Townsend Deprivation Index, smoking status, pack-years of smoking,

alcohol consumption frequency, healthy diet score, physical activity, self-reported health, blood pressure medication, insulin medication, cholesterol lowering medication, genotyping batch, and the first 10 genetic principal components. AAA indicates abdominal aortic aneurysm; CI, confidence interval; HR, hazard ratio.

Supplemental Tables

Table S1. Diagnosis codes used by the UK Biobank for determining the outcome and related medical history.

	ICD-10 codes	OPCS-4 codes	Field ID
**Outcome**			
Abdominal aortic aneurysm	I71.3, I71.4[a]	L18*, L19*, L254, L27*, L28*, L464[b]	41270, 40001-40002, 41272
**Medical history**			
Vascular diseases	I71-I73, I77-I79	/	131382-131387, 131390-131395
Hypertension[c]	I10-I15	/	131286-131295
Diabetes[c]	E10-E14	/	130706-130715
Dyslipidaemia[c]	E78	/	130814-130815
Self-reported aortic aneurysm/dissection, cerebral aneurysm[d]	/	/	20002

ICD indicates International Classification of Disease; OPCS, the Office of Population, Censuses and Surveys: Classification of interventions and Procedures codes.

[a]I71.3 - abdominal aortic aneurysm, ruptured; I71.4 - abdominal aortic aneurysm, without mention of rupture.

[b]L18* - Emergency replacement of aneurysmal segment of aorta; L19* - Other replacement of aneurysmal segment of aorta; L254 - Operations on aneurysm of aorta NEC; L27* - Transluminal insertion of stent graft for aneurysmal segment of aorta; L28* - Transluminal operations on aneurysmal segment of aorta; L464 - Operations on aneurysm of visceral branch of abdominal aorta NEC.

[c]For stratified analyses, cardiometabolic factors are consist of hypertension, diabetes, and dyslipidaemia.

[d]Non-cancer illness code used for aortic aneurysm, aortic dissection, or cerebral aneurysm: 1492 - Aortic aneurysm; 1591 - Aortic aneurysm rupture; 1592 - Aortic dissection; 1425 - Cerebral aneurysm.

Table S2. Definition of a healthy diet score used in this study in the UK Biobank.

Items	Goal (1 point)	One serving equals to	Field ID
Fruits	≥3 servings/day	1 piece of fresh fruit<br>5 pieces of dried fruits	1309, 1319
Vegetables (excluding potatoes)	≥3 servings/day	3 heaped tablespoons	1289, 1299
Whole grains	≥3 servings/day	1 slice of whole-grain bread<br>1 cup of whole-grain cereal	1438, 1448, 1458, 1468
Vegetable oil	≥2 servings/day	Vegetable oil based spread (Flora Pro-Active/Benecol, Soft (tub) margarine, Olive oil based spread, or Polyunsaturated/sunflower oil based spread) in combination with eating at least 2 slices of bread	1428, 2654, 1438
Fish	≥2 servings/week	Once/week	1329, 1339
Dairy	≥2 servings/day	1 cup/day if consumption any type of milk<br>1 piece of cheese	1408, 1418
Refined grains	≤2 servings/day	1 slice of bread or 1 bowl of cereal	1438, 1448, 1458, 1468
Unprocessed meats	≤2 servings/week	Once/week (including poultry, beef, lamb, and pork)	1359, 1369, 1379, 1389
Processed meats	≤1 servings/week	Once/week	1349
Sugar-sweetened beverages	Don't drink	Only 0 serving was possible here	6144

Individuals who met the intake goal for each item were deemed to have adequate intake and awarded one point. These points were then added up to determine the final healthy diet score. A higher score indicates a healthier diet pattern.

Table S3. Data field ID of variables used in the study in the UK Biobank.

Variables	Field ID
**Components of biological ages**	
Forced expiratory volume in 1-second (FEV1)	3063-0.0, 3063-0.1, 3063-0.2
Systolic blood pressure	4080-0.0, 4080-0.1, 93-0.0, 93-0.1
Cholesterol	30690-0.0
Glycated hemoglobin (HbA1c)	30750-0.0
Blood urea nitrogen	30670-0.0
Lymphocyte percentage	30180-0.0
Mean cell volume	30270-0.0
Serum glucose	30740-0.0
Red blood cell (erythrocyte) distribution width	30070-0.0
White blood cell (leukocyte) count	30000-0.0
Albumin	30600-0.0
Creatinine	30700-0.0
C-reactive protein	30710-0.0
Alkaline phosphatase	30610-0.0
**Covariates**	
Age	21022-0.0
Sex	31-0.0
Ethnicity	21000-0.0
Body mass index (BMI)	21001-0.0
Education	6138-0.0
Employment	6142-0.0
Townsend Deprivation Index	22189-0.0
Smoking status	20116-0.0
Pack years of smoking	20161-0.0
Alcohol consumption frequency	1558-0.0
Physical activity	6164-0.0
Overall health rating	2178-0.0
Blood pressure medication	6153-0.0, 6177-0.0
Insulin medication	6153-0.0, 6177-0.0
Cholesterol lowering medication	6153-0.0, 6177-0.0
Genetic principal components	22009-0.1~22009-0.10
Genotype measurement batch	22000-0.0

Table S4. Range of biological age accelerations.

Biological age acceleration	Mean (SD)	Q1	Q2	Q3	Q4
KDMAge acceleration	-0.004 (2.386)	[-10.78, -1.61]	(-1.61, -0.06]	(-0.06, 1.54]	(1.54, 16.23]
PhenoAge acceleration	-0.019 (5.045)	[-16.22, -3.51]	(-3.51, -0.44]	(-0.44, 3.01]	(3.01, 33.46]

KDMAge indicates biological age calculated by the Klemera-Doubal method; PhenoAge, phenotypic age; Q, quartile; SD, standard deviation.

Table S5. Stratified analyses of the associations between biological ageing and the risk of incident abdominal aortic aneurysm.

Subgroups	KDMAge, accelerated ageing		PhenoAge, accelerated ageing	
	HRs (95% CI)	*P* for interaction	HRs (95% CI)	*P* for interaction
Age				
≥60	**1.31 (1.17, 1.46)**	0.968	**1.63 (1.45, 1.84)**	0.912
<60	1.21 (0.98, 1.50)		**1.54 (1.23, 1.93)**	
Sex				
Male	**1.26 (1.13, 1.40)**	0.165	**1.60 (1.43, 1.80)**	0.453
Female	**1.48 (1.16, 1.89)**		**1.78 (1.41, 2.26)**	
BMI, kg/m^2				
≤25	**1.57 (1.28, 1.92)**	**0.004**	**1.98 (1.60, 2.44)**	**<0.001**
25-30	**1.23 (1.07, 1.41)**		**1.74 (1.50, 2.02)**	
≥30	1.14 (0.95, 1.36)		1.16 (0.96, 1.41)	
Smoking status				
Current or former	**1.37 (1.22, 1.53)**	**0.024**	**1.71 (1.52, 1.93)**	0.144
Never	1.04 (0.85, 1.28)		**1.38 (1.13, 1.70)**	
Education				
Degree-level	**1.41 (1.13, 1.75)**	0.304	**1.72 (1.38, 2.16)**	0.831
Non-college	**1.26 (1.13, 1.41)**		**1.61 (1.43, 1.80)**	
Hypertension				
Yes	**1.20 (1.05, 1.38)**	0.114	**1.62 (1.39, 1.88)**	0.563
No	**1.35 (1.18, 1.55)**		**1.63 (1.42, 1.88)**	
Diabetes				
Yes	**1.57 (1.08, 2.27)**	0.431	**1.66 (1.08, 2.54)**	0.748
No	**1.27 (1.15, 1.41)**		**1.64 (1.48, 1.83)**	
Dyslipidaemia				
Yes	**1.25 (1.06, 1.48)**	0.578	**1.58 (1.33, 1.89)**	0.339
No	**1.31 (1.16, 1.48)**		**1.67 (1.47, 1.90)**	

HRs and 95% CIs in bold indicates statistical significance at $P < 0.05$. The covariate used for stratification was removed from the model. Models were adjusted for age, sex, ethnicity, body mass index, education, employment, Townsend Deprivation Index, smoking status, pack-years of smoking, alcohol consumption frequency, healthy diet score, physical activity, self-reported health, blood pressure medication, insulin

medication, cholesterol lowering medication. BMI indicates body mass index; CI, confidence interval; HR, hazard ratio.

Table S6. Association between PRS category and incident abdominal aortic aneurysm risk.

Genetic risk	No. cases/person years	Model 1		Model 2	
		HR (95% CI)	*P* value	HR (95% CI)	*P* value
PRS categories					
Low PRS	478/1,518,654	Ref.		Ref.	
Intermediate PRS	616/1,480,560	1.33 (1.18, 1.50)	<0.001	1.33 (1.17, 1.50)	<0.001
High PRS	754/1,425,718	1.72 (1.53, 1.93)	<0.001	1.69 (1.51, 1.90)	<0.001
P for trend			<0.001		<0.001

Model 1 was adjusted for age, sex, body mass index, education, employment, and Townsend deprivation index.

Model 2 was adjusted for covariates in Model 1 plus smoking status, pack-years of smoking, alcohol consumption frequency, healthy diet score, physical activity, self-reported health, blood pressure medication, insulin medication, cholesterol lowering medication, genotyping batch, and the first 10 genetic principal components.

CI indicates confidence interval; HR, hazard ratio; PRS, polygenic risk score.

Table S7. Association between pack-years of smoking and incident abdominal aortic aneurysm risk.

**Pack-years of smoking**	**HR (95% CI)**	***P* value**
Model 1	1.022 (1.020, 1.023)	<0.001
Model 2	1.020 (1.019, 1.022)	<0.001

Model 1 was adjusted for age, sex, ethnicity, body mass index, education, employment, and Townsend deprivation index.

Model 2 was adjusted for covariates in Model 1 plus smoking status, pack-years of smoking, alcohol consumption frequency, healthy diet score, physical activity, self-reported health, blood pressure medication, insulin medication, cholesterol lowering medication.

CI indicates confidence interval; HR, hazard ratio.

Table S8. Association between pack-years of smoking and biological age acceleration.

Pack-years of smoking	KDMAge acceleration		PhenoAge acceleration	
	β (95% CI)	*P* value	β (95% CI)	*P* value
Model 1	0.015 (0.014, 0.016)	<0.001	0.047 (0.046, 0.048)	<0.001
Model 2	0.013 (0.012, 0.014)	<0.001	0.040 (0.039, 0.042)	<0.001

Model 1 was adjusted for age, sex, ethnicity, body mass index, education, employment, and Townsend deprivation index.

Model 2 was adjusted for covariates in Model 1 plus smoking status, pack-years of smoking, alcohol consumption frequency, healthy diet score, physical activity, self-reported health, blood pressure medication, insulin medication, cholesterol lowering medication.

CI indicates confidence interval; HR, hazard ratio.

Table S9. Mediation effect of biological age acceleration on the association between pack-years of smoking and abdominal aortic aneurysm.

Biological age acceleration	Direct association β (95% CI)	Indirect association β (95% CI)	Mediation proportion % (95% CI)
**Model 1**			
KDMAge	7.86e-05 (7.32e-05, 8.39e-05)	3.34e-06 (2.26e-06, 4.39e-06)	4.08 (2.74, 5.32)
PhenoAge	7.38e-05 (6.83e-05, 7.94e-05)	9.52e-06 (7.80e-06, 1.10e-05)	11.42 (9.43, 13.40)
**Model 2**			
KDMAge	7.46e-05 (6.93e-05, 7.99e-05)	2.58e-06 (1.62e-06, 3.61e-06)	3.34 (2.08, 4.78)
PhenoAge	7.11e-05 (6.55e-05, 7.57e-05)	7.42e-06 (5.94e-06, 8.69e-06)	9.45 (7.67, 11.1)

All *P* value < 0.001. 95% confidence intervals were generated from 1000 bootstrap samples.

Model 1 was adjusted for age, sex, ethnicity, body mass index, education, employment, and Townsend deprivation index.

Model 2 was adjusted for covariates in Model 1 plus smoking status, pack-years of smoking, alcohol consumption frequency, healthy diet score, physical activity, self-reported health, blood pressure medication, insulin medication, cholesterol lowering medication.

CI indicates confidence interval.

Table S10. Association between biological age acceleration and incident abdominal aortic aneurysm risk after excluding participants diagnosed with AAA during the first two years of follow-up.

	Hazard ratio (95% CI)	*P* value	*P* for trend
**KDMAge acceleration**			
Quartile 1	Ref.		<0.001
Quartile 2	1.22 (1.06, 1.41)	0.007	
Quartile 3	1.29 (1.12, 1.49)	<0.001	
Quartile 4	1.54 (1.34, 1.78)	<0.001	
Continuous, per SD increase	1.06 (1.04, 1.08)	<0.001	
Non-accelerated ageing[a]	Ref.		
Accelerated ageing[b]	1.27 (1.15, 1.40)	<0.001	
**PhenoAge acceleration**			
Quartile 1	Ref.		<0.001
Quartile 2	1.29 (1.08, 1.55)	0.005	
Quartile 3	1.47 (1.24, 1.75)	<0.001	
Quartile 4	2.26 (1.91, 2.67)	<0.001	
Continuous, per SD increase	1.06 (1.05, 1.07)	<0.001	
Non-accelerated ageing[a]	Ref.		
Accelerated ageing[b]	1.57 (1.41, 1.74)	<0.001	

Model was adjusted for age, sex, ethnicity, body mass index, education, employment, Townsend Deprivation Index, smoking status, pack-years of smoking, alcohol consumption frequency, healthy diet score, physical activity, self-reported health, blood pressure medication, insulin medication, cholesterol lowering medication. AAA indicates abdominal aortic aneurysm; CI, confidence interval; KDMAge, biological age calculated by the Klemera-Doubal method; PhenoAge, phenotypic age; SD, standard deviation.

[a]Non-accelerated ageing indicates that the residual of the regression of KDMAge or PhenoAge based on chronological age is ≤0.

[b]Accelerated ageing indicates that the residual of the regression of KDMAge or PhenoAge based on chronological age is >0.

Table S11. Association between biological age acceleration and incident abdominal aortic aneurysm risk in participants without missing covariates.

	Hazard ratio (95% CI)	*P* value	*P* for trend
**KDMAge acceleration**			
Quartile 1	Ref.		<0.001
Quartile 2	1.19 (1.02, 1.40)	0.031	
Quartile 3	1.27 (1.08, 1.48)	0.003	
Quartile 4	1.55 (1.33, 1.81)	<0.001	
Continuous, per SD increase	1.07 (1.04, 1.09)	<0.001	
Non-accelerated ageing[a]	Ref.		
Accelerated ageing[b]	1.28 (1.15, 1.43)	<0.001	
**PhenoAge acceleration**			
Quartile 1	Ref.		<0.001
Quartile 2	1.30 (1.07, 1.59)	0.009	
Quartile 3	1.48 (1.22, 1.80)	<0.001	
Quartile 4	2.35 (1.96, 2.83)	<0.001	
Continuous, per SD increase	1.06 (1.05, 1.08)	<0.001	
Non-accelerated ageing[a]	Ref.		
Accelerated ageing[b]	1.60 (1.43, 1.80)	<0.001	

Model was adjusted for age, sex, ethnicity, body mass index, education, employment, Townsend Deprivation Index, smoking status, pack-years of smoking, alcohol consumption frequency, healthy diet score, physical activity, self-reported health, blood pressure medication, insulin medication, cholesterol lowering medication. CI indicates confidence interval; KDMAge, biological age calculated by the Klemera-Doubal method; PhenoAge, phenotypic age; SD, standard deviation.

[a]Non-accelerated ageing indicates that the residual of the regression of KDMAge or PhenoAge based on chronological age is ≤0.

[b]Accelerated ageing indicates that the residual of the regression of KDMAge or PhenoAge based on chronological age is >0.

Table S12. Association between biological age acceleration and incident abdominal aortic aneurysm risk among participants without self-reported poor health at baseline.

	**Hazard ratio (95% CI)**	***P* value**	***P* for trend**
**KDMAge acceleration**			
Quartile 1	Ref.		<0.001
Quartile 2	1.27 (1.09, 1.46)	0.002	
Quartile 3	1.33 (1.15, 1.54)	<0.001	
Quartile 4	1.58 (1.37, 1.82)	<0.001	
Continuous, per SD increase	1.07 (1.04, 1.09)	<0.001	
Non-accelerated ageing[a]	Ref.		
Accelerated ageing[b]	1.30 (1.17, 1.43)	<0.001	
**PhenoAge acceleration**			
Quartile 1	Ref.		<0.001
Quartile 2	1.25 (1.04, 1.50)	0.016	
Quartile 3	1.50 (1.26, 1.78)	<0.001	
Quartile 4	2.29 (1.94, 2.71)	<0.001	
Continuous, per SD increase	1.07 (1.05, 1.08)	<0.001	
Non-accelerated ageing[a]	Ref.		
Accelerated ageing[b]	1.61 (1.45, 1.79)	<0.001	

Model was adjusted for age, sex, ethnicity, body mass index, education, employment, Townsend Deprivation Index, smoking status, pack-years of smoking, alcohol consumption frequency, healthy diet score, physical activity, self-reported health, blood pressure medication, insulin medication, cholesterol lowering medication. CI indicates confidence interval; KDMAge, biological age calculated by the Klemera-Doubal method; PhenoAge, phenotypic age; SD, standard deviation.

[a]Non-accelerated ageing indicates that the residual of the regression of KDMAge or PhenoAge based on chronological age is ≤0.

[b]Accelerated ageing indicates that the residual of the regression of KDMAge or PhenoAge based on chronological age is >0.

Table S13. Association between biological age acceleration and incident abdominal aortic aneurysm risk in the Fine and Gray competing risk model.

Biological age acceleration	Number of AAA events	Number of competing events	SHRs (95% CIs) for continuous (per SD increase)	*P* value
KDMAge acceleration	1886	27856	1.04 (1.02, 1.07)	<0.001
PhenoAge acceleration	1886	27856	1.05 (1.04, 1.06)	<0.001

Fine and Gray model was adjusted for age, sex, ethnicity, body mass index, education, employment, Townsend Deprivation Index, smoking status, pack-years of smoking, alcohol consumption frequency, healthy diet score, physical activity, self-reported health, blood pressure medication, insulin medication, cholesterol lowering medication. CI indicates confidence interval; KDMAge, biological age calculated by the Klemera-Doubal method; PhenoAge, phenotypic age; SD, standard deviation; SHRs, sub-distribution hazard ratios.
