# Supplementary material for: Associations of biological ageing and genetic risk with incident abdominal aortic aneurysm
Source: Commun Med (Lond). 2026 Jan 9;6:107. doi: 10.1038/s43856-025-01373-w (PMC12894926; doi:10.1038/s43856-025-01373-w)
Supplement: Supplementary file 3 — Description of Additional Supplementary Data [file 43856_2025_1373_MOESM3_ESM.docx]

Description of additional supplementary file

File name: Supplementary Data 1

Description: Summary information of 31 independent SNPs for weighted polygenic risk score construction.

File name: Supplementary Data 2

Description: Baseline characteristics of the participants included in the study.

File name: Supplementary Data 3

Description: Baseline characteristics of white Europeans included in the study.

File name: Supplementary Data 4

Description: Hazard ratios for incident abdominal aortic aneurysm risk from full Cox models including biological age acceleration and covariates.

File name: Supplementary Data 5

Description: Restricted cubic spline estimates of the dose-response association between biological age accelerations and incident abdominal aortic aneurysm risk.

File name: Supplementary Data 6

Description: Estimated hazard ratios (HRs) with 95% confidence intervals for the joint association of biological age acceleration and genetic predisposition with incident abdominal aortic aneurysm risk.
